# Supplementary material for: Natural variation of STKc_GSK3 kinase TaSG-D1 contributes to heat stress tolerance in Indian dwarf wheat
Source: Nat Commun. 2024 Mar 7;15:2097. doi: 10.1038/s41467-024-46419-0 (PMC10920922; doi:10.1038/s41467-024-46419-0)
Supplement: Supplementary file 1 — Supplementary information [file 41467_2024_46419_MOESM1_ESM.pdf]

Natural variation of STKc_GSK3 kinase TaSG-D1 contributes to heat stress tolerance in Indian dwarf wheat

Cao *et al*.

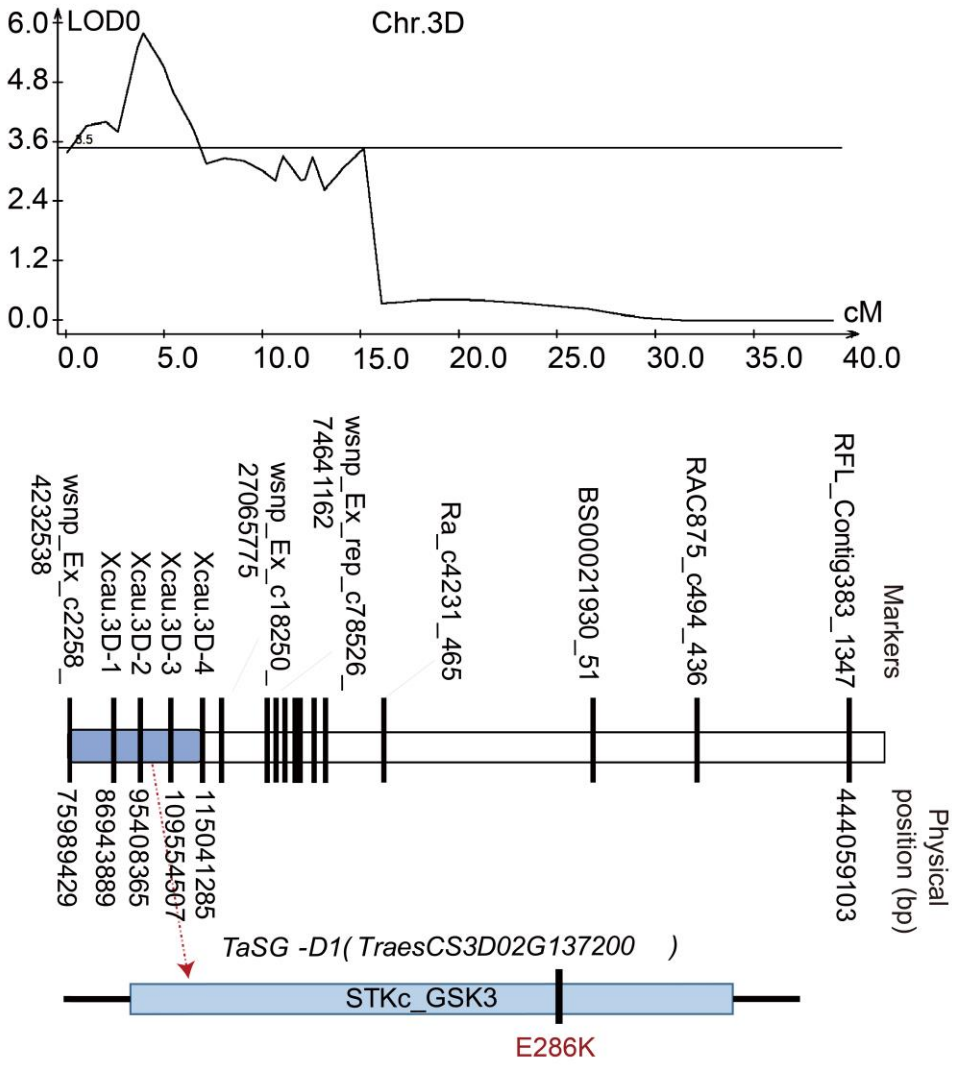

Supplementary Figure 1. QTL mapping. QTL mapping using an F_7 recombinant inbred line population derived from ND4332 and HS2. A candidate QTL is identified in the region between wsnp_Ex_c2258_4232538 and Xcau.3D-4 on the short arm of chromosome 3D. *TaSG-D1*^{E286K} harbors an E286K amino acid substitution. Source data are provided as a Source Data file.

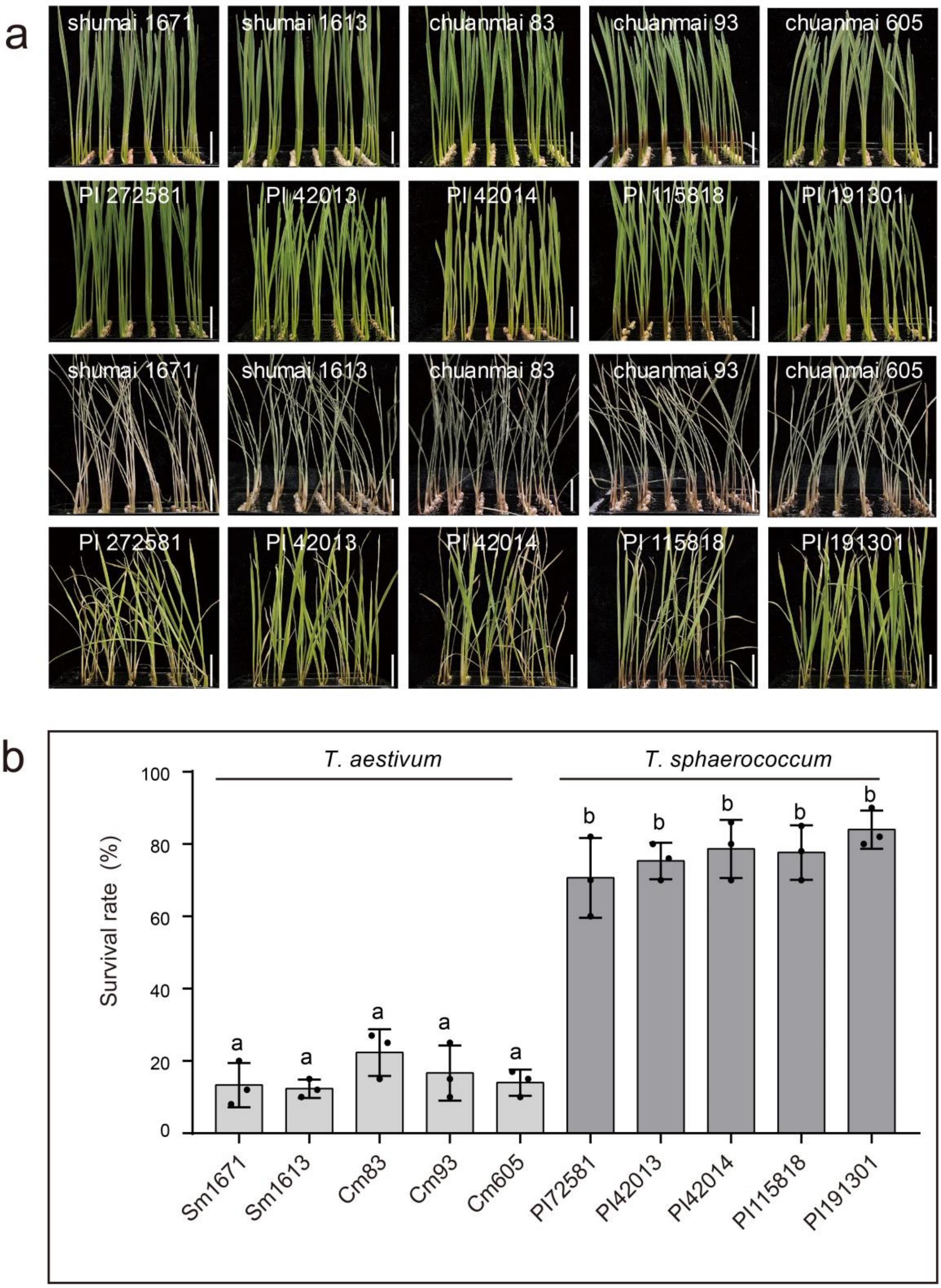

Supplementary Figure 2. ***T. sphaerococcum*** **exhibits enhanced heat tolerance compared to** ***T. aestivum*****.** **(a)** Heat tolerance assay (42°C for 2 days) of five *T. sphaerococcum* and five *T. aestivum* accessions. Bar, 2 cm. **(b)** Plant survival rates in response to heat stress. Statistical analysis was performed with GraphPad Prism 7 (v7.00). Each bar represents standard deviation, columns labeled with different alphabet were considered significantly different ($P < 0.05$, one-way ANOVA analysis-Tukey comparison; n = 3 biologically independent experiments. Source data are provided as a Source Data file.

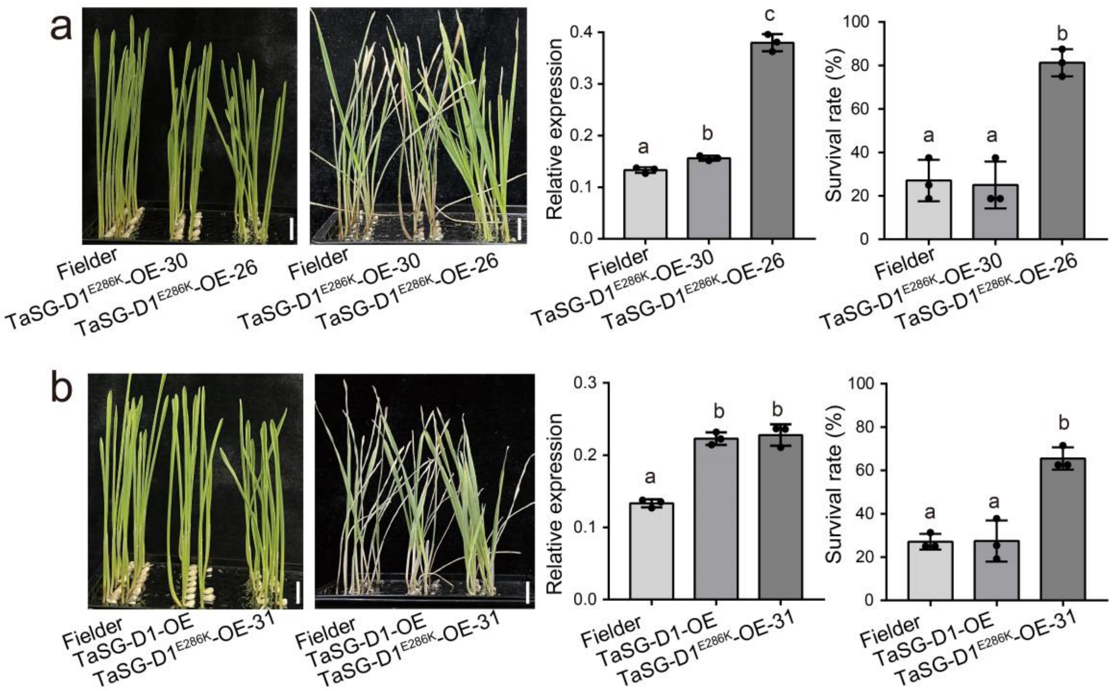

Supplementary Figure 3. Phenotypic and statistical analysis of *TaSG-D1* and *TaSG-D1^{E286K}* overexpression lines in response to heat stress. (a) *TaSG-D1^{E286K}* overexpression lines with higher expression level exhibits enhanced heat tolerance. **(b)** *TaSG-D1^{E286K}* overexpression line shows improved heat tolerance compared to *TaSG-D1* overexpression line with similar expression levels (42°C for 3 days). Scale bars = 1 cm. Statistical analysis was performed with GraphPad Prism 7 (v7.00). Error bars represent standard deviation, columns labeled with different alphabet were considered significantly different ($P < 0.05$, one-way ANOVA analysis-Tukey comparison; n = 3 biologically independent experiments. Source data are provided as a Source Data file.

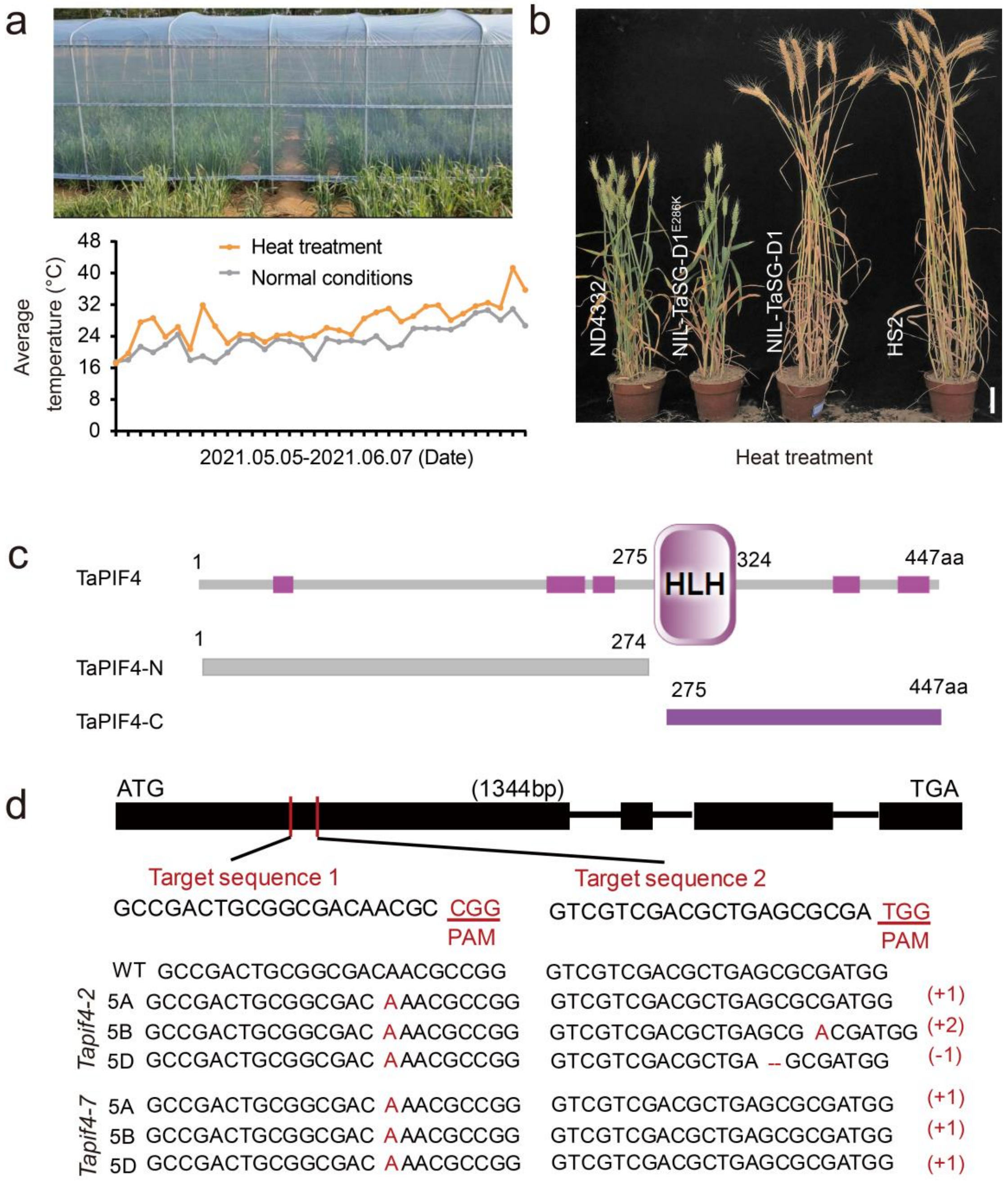

Supplementary Figure 4. NIL$^{TaSG\text{-}D1\text{-}E286K}$ exhibits enhanced heat tolerance compared to NIL$^{TaSG\text{-}D1}$ at adult stage. (a) Plastic tunnel was used for heat treatment of wheat at adult stage (upper), daily average temperature inside and outside the plastic tunnel are shown (lower). Statistical analysis was performed with GraphPad Prism 7 (v7.00). **(b)** Performance of near isogenic lines of NIL$^{TaSG\text{-}D1}$ and NIL$^{TaSG\text{-}D1\text{-}E286K}$ in response to heat stress at adult stage. **(c)** Schematic diagram of the N-terminus of TaPIF4 (1-274aa) and the C-terminus of TaPIF4 (275-447aa) used in the Y2H assay. **(d)** Strategy used to generate the *TaPIF4* knockout mutants. The target sequences of sgRNAs are conserved among the three *TaPIF4* homeologs. The protospacer-adjacent motif (PAM) sequence is highlighted in red letters. The numbers in brackets indicate the mutations caused by genome editing with the two sgRNAs. Source data are provided as a Source Data file.

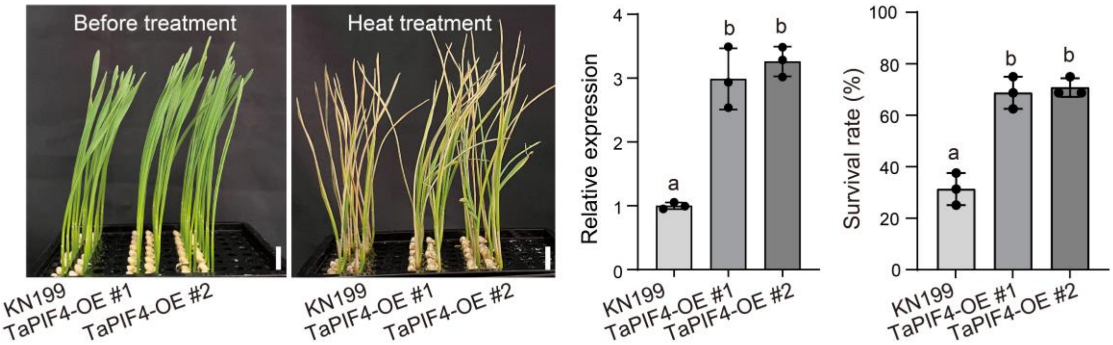

Supplementary Figure 5. Phenotypic and statistical analysis of *TaPIF4* overexpression lines in response to heat stress. *TaPIF4* overexpression contributes to improved heat tolerance compared to WT (KN199, 42°C for 3 days). Scale bars = 1 cm. Statistical analysis was performed with GraphPad Prism 7 (v7.00). Error bars represent standard deviation, columns labeled with different alphabet were considered significantly different ($P < 0.05$, one-way ANOVA analysis-Tukey comparison; n = 3 biologically independent experiments. Source data are provided as a Source Data file.

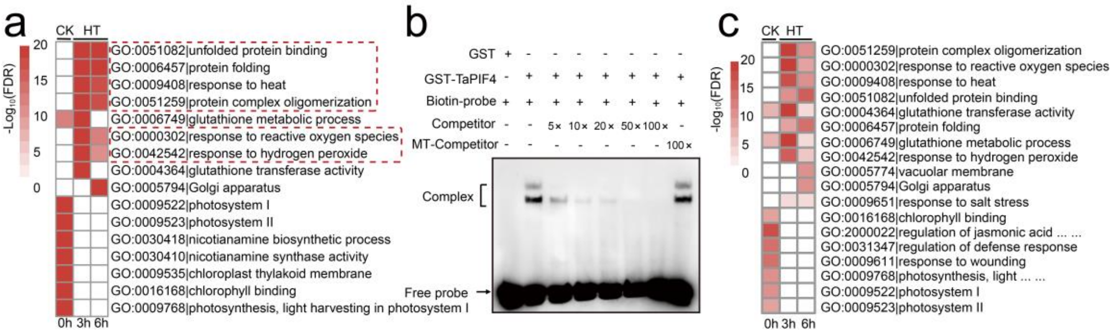

Supplementary Figure 6. Gene Ontology enrichment analysis and validation of *TaPIF4*-dependent heat responsive genes. (a) The heat responsive down-regulated genes in *Tapif4* knockout lines are enriched in “unfolded protein binding”, “protein folding”, “response to heat” and “response to reactive oxygen species” categories. CK, normal conditions; HT, heat treatment**. (b)** The TaPIF4 transcription factor binds to the E-box motif in the *TaMBF1c* promoter region. The “+” and “−” symbols indicate the presence and absence of corresponding probes, or proteins. The terms “5×”, “10×”, “20×”, “50×”, and “100×” of competitor and mutated competitor indicate 5-, 10-, 20-, 50-, and 100-fold molar excess of competitor or mutated competitor probes relative to biotin-labeled probes. Three independent experiments were performed. **(c)** The heat responsive down-regulated genes with TaPIF4 binding motif (E-box motif) in their 1-kb promoter are also enriched in “unfolded protein binding”, “protein folding”, “response to heat” and “response to reactive oxygen species” categories. CK, normal conditions; HT, heat treatment. Source data are provided as a Source Data file.

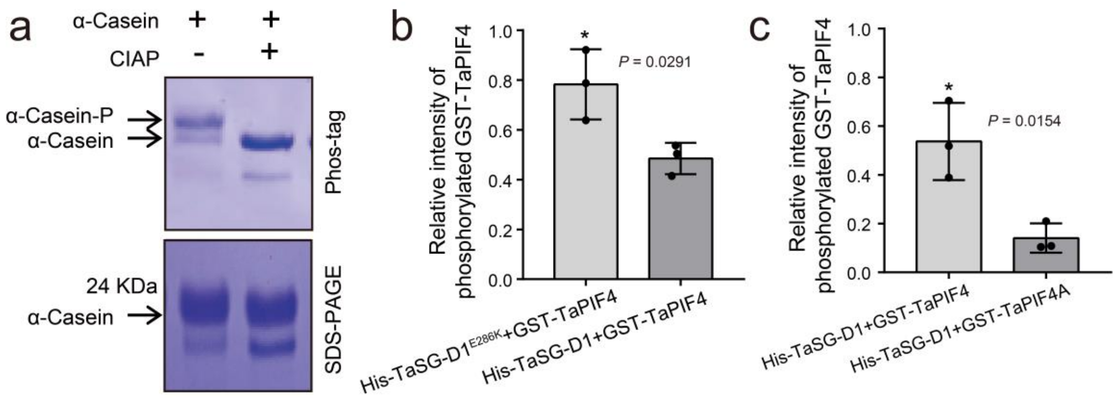

Supplementary Figure 7. TaPIF4 exhibits an increased phosphorylation level when co-expressing with TaSG-D1^{E286K} compared with TaSG-D1. (a) Coomassie brilliant blue (CBB)-staining was carried out on α-casein and dephosphorylated α-casein samples after being subjected to both Phos-tag SDS-PAGE and standard SDS-PAGE to assess the efficiency of the Phos-tag. **(b)** Quantification of phosphorylation intensity of TaPIF4 in conditions of TaSG-D1/ TaSG-D1^{E286K}. Statistical analysis was performed with GraphPad Prism 7 (v7.00). *$P < 0.05$, unpaired two-tailed *t*-test, n = 3 independent experiments. **(c)** Quantification of phosphorylation intensity of TaPIF4 and TaPIF4A in conditions of TaSG-D1. *$P < 0.05$, unpaired two-tailed *t*-test, n = 3 independent experiments. Source data are provided as a Source Data file.

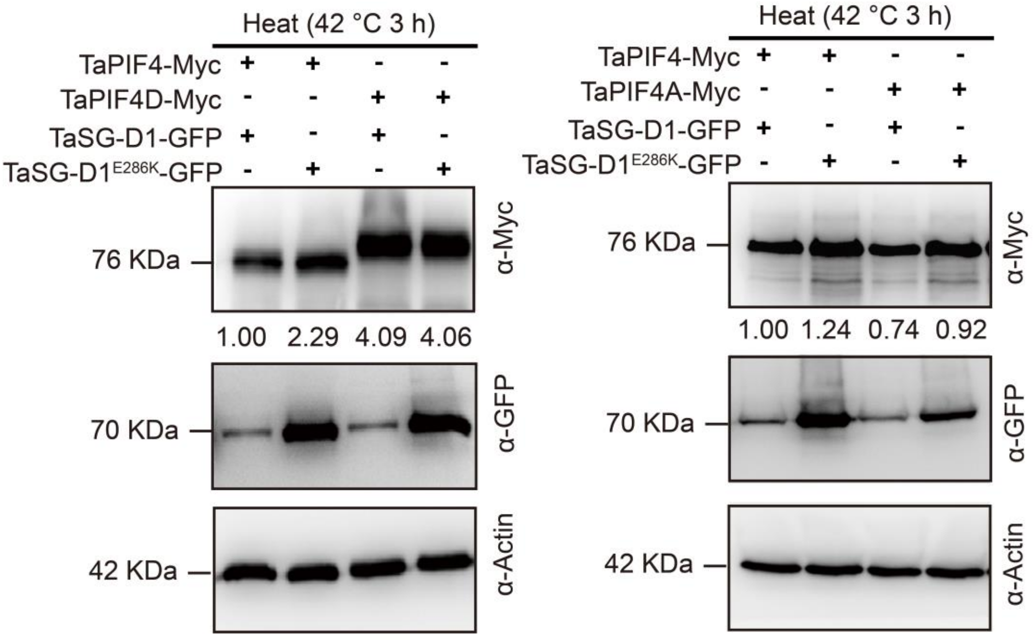

Supplementary Figure 8. Mutation of phosphorylation sites affects the protein stability of TaPIF4. TaSG-D1/TaSG-D1^{E286K} regulate TaPIF4 protein stability via phosphorylation. 35S: TaSG-D1-GFP/TaSG-D1^{E286K}-GFP and 35S: TaPIF4/TaPIF4D/TaPIF4A-Myc were co-infiltrated into *N. benthamiana* leaves, followed by treatment at 42°C for 3 h. The relative protein abundance of TaPIF4 was quantified using ImageJ software. The relative protein level of TaPIF4 at the first column was defined as 1.00. Three independent experiments were performed. Source Data are provided as a Source Data file.

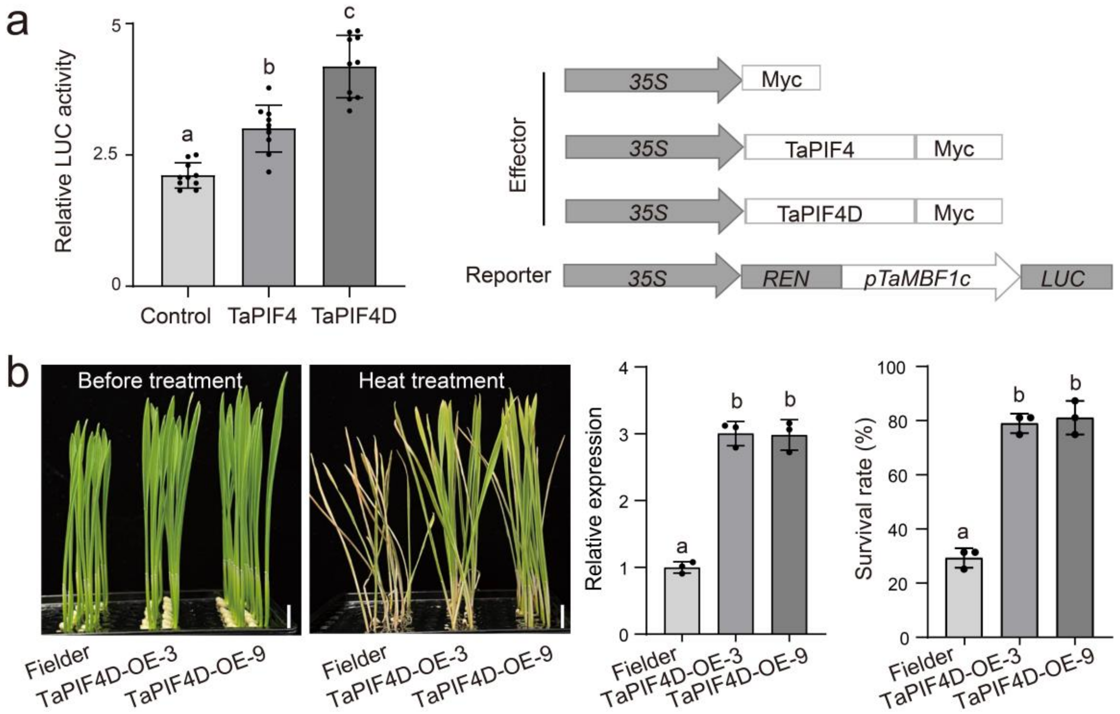

Supplementary Figure 9. Phenotypic and statistical analysis of *TaPIF4D* overexpression lines in response to heat stress. (a) TaPIF4D activates *TaMBF1c* expression more efficiently compared with TaPIF4. The reporter construct *pTaMBF1c: LUC* with the indicated effector constructs *(35S: Myc; 35S:TaPIF4-Myc; 35S:TaPIF4D-Myc*) were co-transformed into *N. benthamiana* leaves, respectively. Relative LUC activity (LUC/REN) reflects transcriptional activity of TaPIF4 and TaPIF4D; Statistical analysis was performed with GraphPad Prism 7 (v7.00). Error bars represent standard deviation, columns labeled with different alphabet were considered significantly different ($P < 0.05$, one-way ANOVA analysis-Tukey comparison; n = 10 biologically independent experiments). **(b)** *TaPIF4D* overexpression improves heat tolerance compared to WT (Fielder, 42°C for 3 days). Scale bars = 1 cm. Statistical analysis was performed with GraphPad Prism 7 (v7.00). Error bars represent standard deviation, columns labeled with different alphabet were considered significantly different ($P < 0.05$, one-way ANOVA analysis-Tukey comparison; n = 3 biologically independent experiments). Source data are provided as a Source Data file.

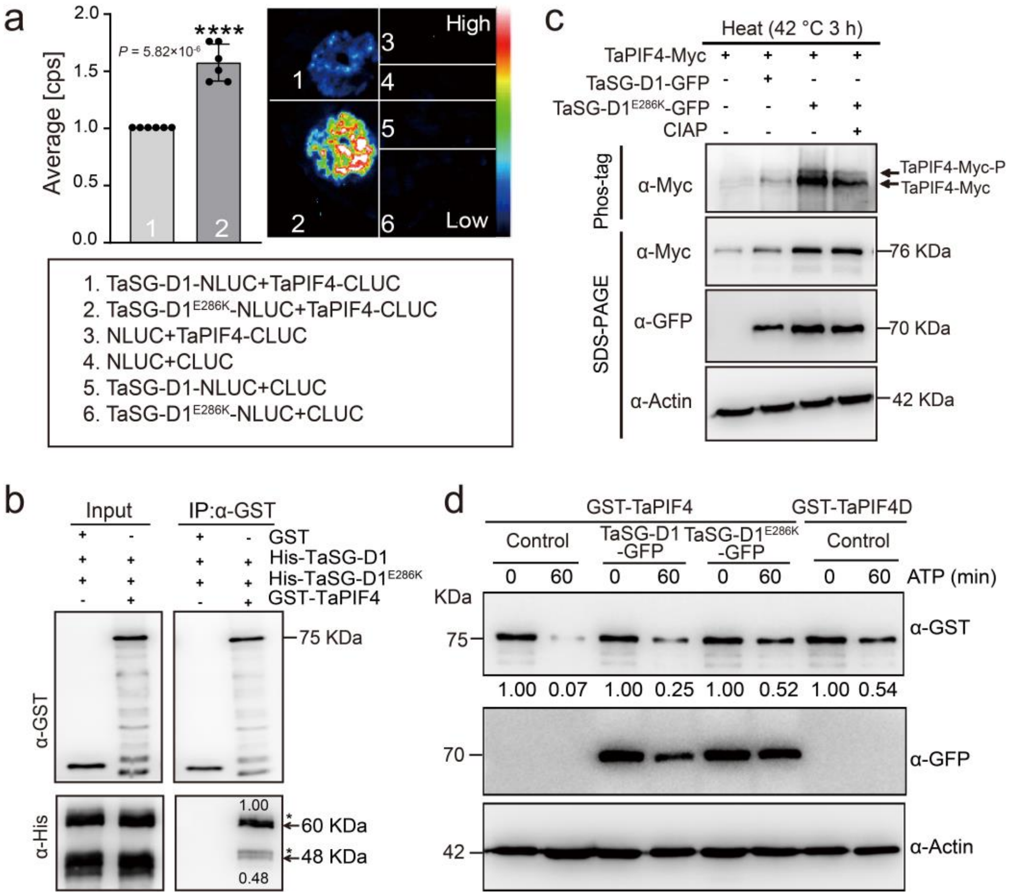

Supplementary Figure 10. TaPIF4 exhibits a stronger interaction and a higher phosphorylation level when co-expressing with TaSG-D1^{E286K} than TaSG-D1. (a) TaSG-D1^{E286K} exhibits a stronger interaction with TaPIF4 compared to TaSG-D1 in an LCI assay in *N. benthamiana* leaves. The average counts [cps] was calculated with Indigo software (v2.0.5.0). Statistical analysis was performed with GraphPad Prism 7 (v7.00). Each bar represents standard deviation. ****$P < 0.0001$ (n = 6 biologically independent experiments; unpaired two-tailed *t*-test). **(b)** TaSG-D1^{E286K} exhibits a stronger interaction with TaPIF4 compared to TaSG-D1 in a pull-down assay. GST/GST-TaPIF4 were incubated with His-TaSG-D1 (48 KDa) and His-TaSG-D1^{E286K} (60 KDa). Protein mixture was immunoprecipitated with Proteinlso GST Resin and detected with anti-His (lower panel) and anti-GST (upper panel) antibodies. The arrows in the lower panel indicates non-phosphorylated TaSG-D1 (48 KDa) and TaSG-D1^{E286K} (60 KDa), respectively. "*" in the lower panel indicates self-phosphorylated forms of TaSG-D1 and TaSG-D1^{E286K}. Three independent experiments were performed. **(c)** The phosphorylation level of TaPIF4 is higher when co-expressing with *TaSG-D1^{E286K}* than with *TaSG-D1* in *N. benthamiana* leaves, as revealed in a Phos-tag gel assay. The phosphorylation status of TaPIF4 is presented in the upper panel (anti-Myc). The protein abundances of TaPIF4 (second panel, anti-Myc) and TaSG-D1/TaSG-D1^{E286K} (third panel, anti-GFP) are shown in the SDS-PAGE gels. Actin was detected with anti-Actin antibody as a loading control (bottom panel). Three independent experiments were performed. **(d)** TaSG-D1^{E286K} attenuates TaPIF4/TaPIF4D degradation in 42°C cell-free degradation assays corresponding to **Fig. 4b**. The relative protein abundance of TaPIF4/TaPIF4D was quantified using ImageJ software. The relative protein level of TaPIF4/TaPIF4D at 0 was defined as 1.00. Three independent experiments were performed. Source data are provided as a Source Data file.

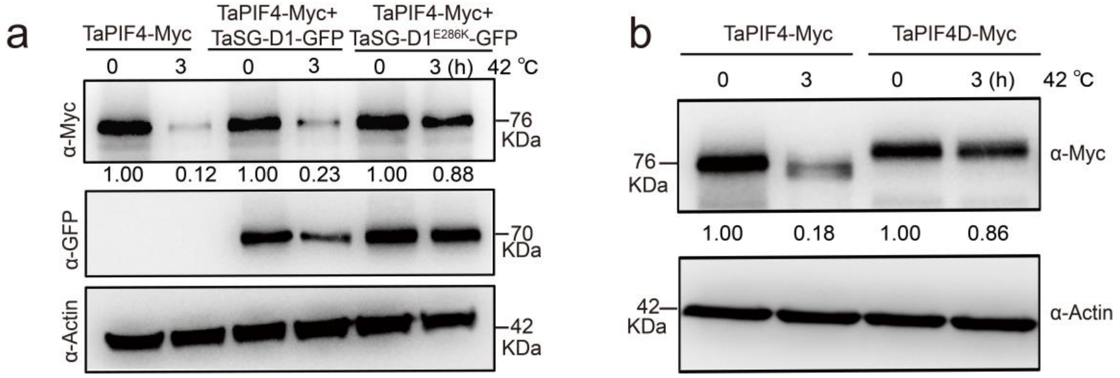

Supplementary Figure 11. TaSG-D1/TaSG-D1^{E286K} regulate TaPIF4 protein stability under heat stress treatment. (a) TaPIF4 protein exhibits less degraded levels in the presence of TaSG-D1 and TaSG-D1^{E286K}. 35S: TaSG-D1-GFP/TaSG-D1^{E286K}-GFP and 35S: TaPIF4 -Myc were infiltrated into *N. benthamiana* leaves, respectively, followed by treatment at 42°C for 3 h. The relative protein abundance of TaPIF4 was quantified using ImageJ software. The relative protein level of TaPIF4 at each control conditions was defined as 1.00. Three independent experiments were performed. **(b)** TaPIF4D protein exhibits reduced degradation compared with TaPIF4 under heat stressed conditions. 35S: TaPIF4 -Myc and 35S: TaPIF4D-Myc were infiltrated into *N. benthamiana* leaves, respectively, followed by treatment at 42°C for 3 h. The relative protein abundance of TaPIF4 or TaPIF4D was quantified using ImageJ software. The relative protein level of TaPIF4 or TaPIF4D at each control conditions was defined as 1.00. Three independent experiments were performed. Source data are provided as a Source Data file.

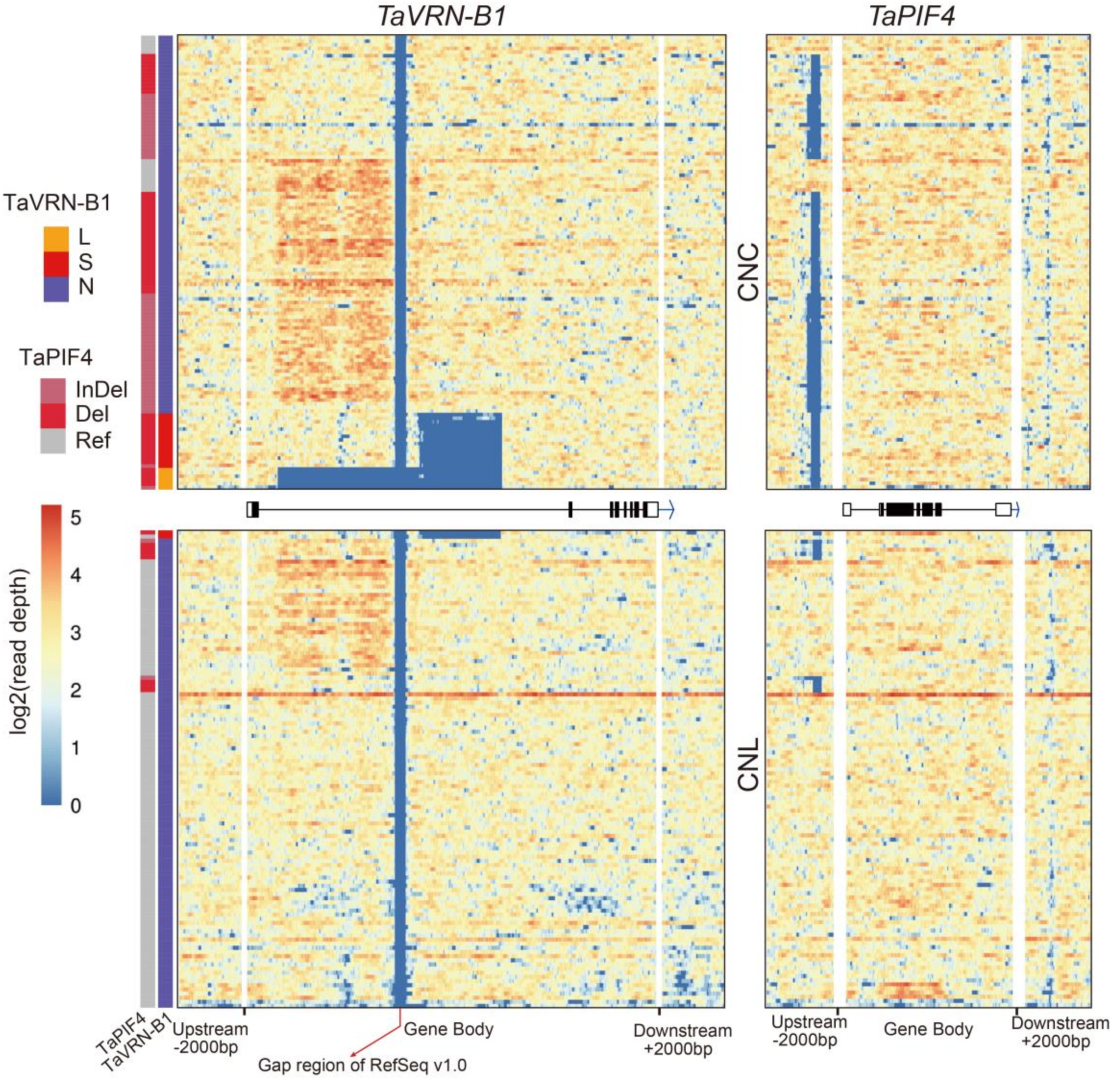

Supplementary Figure 12. Genomic differentiation of *TaPIF4* and *TaVrn-B1* between Chinese landraces and cultivars. Heat maps of read depth for *TaVrn-B1* and *TaPIF4* including gene body and 2000-bp flanking sequence. L and S refer to the two haplotypes that with ~6800 bp and ~2500 bp deletion, respectively, in the first intron of *TaVrn-B1* and N represents the reference haplotype. CNC and CNL represent Chinese cultivated varieties (n = 125) and landraces varieties (n = 116), respectively.
