# Supplementary material for: Natural variation of STKc_GSK3 kinase TaSG-D1 contributes to heat stress tolerance in Indian dwarf wheat
Source: Nat Commun. 2024 Mar 7;15:2097. doi: 10.1038/s41467-024-46419-0 (PMC10920922; doi:10.1038/s41467-024-46419-0)
Supplement: Supplementary file 3 — Description of Additional Supplementary Files [file 41467_2024_46419_MOESM3_ESM.pdf]

Description of Additional Supplementary Files

File Name: Supplementary Data 1
Description: Annotated genes within the genetic interval between markers of wsnp Ex c2258_4232538 and·Xcau.3D-4.

File Name: Supplementary Data 2
Description: Candidates of TaSG-D1-interacting protein from Y2H screening.

File Name: Supplementary Data 3
Description: Down-regulated genes in Tapif4 knockout lines compared with WT at 0 h, 3 h and 6 h after heat stress, respectively.

File Name: Supplementary Data 4
Description: LC-MS/MS results of TaPIF4 phosphorylation sites analysis.

File Name: Supplementary Data 5
Description: Passport information and haplotype of 331 hexaploid wheat accessions in this study.

File Name: Supplementary Data 6
Description: Primers used in this study.
