# Supplementary material for: Natural variation of STKc_GSK3 kinase TaSG-D1 contributes to heat stress tolerance in Indian dwarf wheat
Source: Nat Commun. 2024 Mar 7;15:2097. doi: 10.1038/s41467-024-46419-0 (PMC10920922; doi:10.1038/s41467-024-46419-0)
Supplement: Supplementary file 10 — Reporting Summary [file 41467_2024_46419_MOESM10_ESM.pdf]

nature portfolio

Corresponding author(s): Mingming Xin

Last updated by author(s): Feb 19, 2024

Reporting Summary

Nature Portfolio wishes to improve the reproducibility of the work that we publish. This form provides structure for consistency and transparency in reporting. For further information on Nature Portfolio policies, see our Editorial Policies and the Editorial Policy Checklist.

Statistics

For all statistical analyses, confirm that the following items are present in the figure legend, table legend, main text, or Methods section.

n/a	Confirmed	
☐	☒	The exact sample size (*n*) for each experimental group/condition, given as a discrete number and unit of measurement
☐	☒	A statement on whether measurements were taken from distinct samples or whether the same sample was measured repeatedly
☐	☒	The statistical test(s) used AND whether they are one- or two-sided<br>*Only common tests should be described solely by name; describe more complex techniques in the Methods section.*
☒	☐	A description of all covariates tested
☐	☒	A description of any assumptions or corrections, such as tests of normality and adjustment for multiple comparisons
☐	☒	A full description of the statistical parameters including central tendency (e.g. means) or other basic estimates (e.g. regression coefficient) AND variation (e.g. standard deviation) or associated estimates of uncertainty (e.g. confidence intervals)
☐	☒	For null hypothesis testing, the test statistic (e.g. *F*, *t*, *r*) with confidence intervals, effect sizes, degrees of freedom and *P* value noted<br>*Give P values as exact values whenever suitable.*
☒	☐	For Bayesian analysis, information on the choice of priors and Markov chain Monte Carlo settings
☒	☐	For hierarchical and complex designs, identification of the appropriate level for tests and full reporting of outcomes
☒	☐	Estimates of effect sizes (e.g. Cohen's *d*, Pearson's *r*), indicating how they were calculated

Our web collection on statistics for biologists contains articles on many of the points above.

Software and code

Policy information about availability of computer code

Data collection	Indigo (v2.0.5.0) was used to collect Luminescence and average counts [cps]. SparkControl (v2.1) was used for transient dual luciferase (dual-LUC) assay. Elitech(RC-4) and ElitechLog (v6.4.0) was used to collect temperature data. For RNA-seq, the library products were sequenced in an Illumina Novaseq 6000 platform.
Data analysis	Softwares used in this study include the followings: WinQTLCart (v2.5), GraphPad Prism (v7.00), Image J (v1.52i), Fastp (v.0.19.4), kallisto (v.0.45.0), R language (v3.6.0), R package ClusterProfiler (v.3.12.0), and DEseq2 (v.1.24.0), BWA-MEM (0.7.15-r1140), VCFtools (v0.1.13), Bamtools (v2.4.1), Samtools (v1.3.1), GATK (v3.8), fimo (v. 4.11.2). Custom codes (https://github.com/QinZhen1995/CAU-TaSG).

For manuscripts utilizing custom algorithms or software that are central to the research but not yet described in published literature, software must be made available to editors and reviewers. We strongly encourage code deposition in a community repository (e.g. GitHub). See the Nature Portfolio guidelines for submitting code & software for further information.

Data

Policy information about availability of data

All manuscripts must include a data availability statement. This statement should provide the following information, where applicable:

- Accession codes, unique identifiers, or web links for publicly available datasets
- A description of any restrictions on data availability
- For clinical datasets or third party data, please ensure that the statement adheres to our policy

The RNA-seq data have been deposited in the NCBI database under accession code PRJNA906524 [https://www.ncbi.nlm.nih.gov/bioproject/PRJNA906524]. The

raw sequence data of previously published re-sequenced accessions used in this study are available in the Sequence Read Archive under accession code PRJNA476679 [https://www.ncbi.nlm.nih.gov/bioproject/PRJNA476679], PRJNA596843 [https://www.ncbi.nlm.nih.gov/bioproject/PRJNA596843], PRJNA439156 [https://www.ncbi.nlm.nih.gov/bioproject/PRJNA439156], PRJNA663409 [https://www.ncbi.nlm.nih.gov/bioproject/PRJNA663409], PRJNA597250 [https://www.ncbi.nlm.nih.gov/bioproject/PRJNA597250]. The raw sequence data of previously published re-sequenced accessions used in this study are available in the Genome Sequence Archive (http://bigd.big.ac.cn/gsa) under accession number CRA001870 [https://ngdc.cncb.ac.cn/gsa/browse/CRA001870] and CRA001951 [https://ngdc.cncb.ac.cn/gsa/browse/CRA001951]. The Chinese Spring wheat reference genome (IWGSC RefSeq v1.0) is publicly available at [https://wheat-urgi.versailles.inra.fr/Seq-Repository/Assemblies]. The UniProt wheat sequence database is publicly available at [https://www.uniprot.org/]. Source data are provided with this paper.

Research involving human participants, their data, or biological material

Policy information about studies with human participants or human data. See also policy information about sex, gender (identity/presentation), and sexual orientation and race, ethnicity and racism.

Reporting on sex and gender	not applicable
Reporting on race, ethnicity, or other socially relevant groupings	not applicable
Population characteristics	not applicable
Recruitment	not applicable
Ethics oversight	not applicable

Note that full information on the approval of the study protocol must also be provided in the manuscript.

Field-specific reporting

Please select the one below that is the best fit for your research. If you are not sure, read the appropriate sections before making your selection.

[x] Life sciences   [ ] Behavioural & social sciences   [ ] Ecological, evolutionary & environmental sciences

For a reference copy of the document with all sections, see nature.com/documents/nr-reporting-summary-flat.pdf

Life sciences study design

All studies must disclose on these points even when the disclosure is negative.

Sample size	Sample size was described in each Figure legend. For phenotype identification, at least three replicates were performed in each condition, each replicate contained 16-48 individual plants according to previous studies in the area of plant science [Tian X, Qin Z, Zhao Y, et al. New Phytol, 2022 (DOI: 10.1111/nph.17865); Tian, G., Wang, S., Wu, J. et al. Nat commun, 2023 (DOI: 10.1038/s41467-023-36901-6); Kan, Y., Mu, XR., Zhang, H. et al. Nat Plants, 2022 (DOI: 10.1038/s41477-021-01039-0)]. We did not use any statistical method to predetermine sample size. 331 re-sequencing data were downloaded from published database as detailed in the Methods.
Data exclusions	No data were excluded from the analyses.
Replication	All experiments were independently and successfully repeated for at least three times.
Randomization	Most of the experimental findings were related to comparative analysis according to the plant genotypes (e.g. wild-type vs. Tapif4 and TaSG-D1 vs. TaSG-D1E286K). Covariates can be neglected because the genetic background is highly similar. For the sampling in the study, we always randomly select individuals within the group.
Blinding	No blinding was used in the study due to all genotypes in our materials were independently labeled. Investigators were not blinded because these kinds of experiments did not genetically need blinding.

Reporting for specific materials, systems and methods

We require information from authors about some types of materials, experimental systems and methods used in many studies. Here, indicate whether each material, system or method listed is relevant to your study. If you are not sure if a list item applies to your research, read the appropriate section before selecting a response.

Materials & experimental systems

n/a	Involved in the study
[ ]	[x] Antibodies
[ ]	[x] Eukaryotic cell lines
[x]	[ ] Palaeontology and archaeology
[x]	[ ] Animals and other organisms
[x]	[ ] Clinical data
[x]	[ ] Dual use research of concern
[ ]	[x] Plants

Methods

n/a	Involved in the study
[x]	[ ] ChIP-seq
[x]	[ ] Flow cytometry
[x]	[ ] MRI-based neuroimaging

Antibodies

Antibodies used

ProteinFind® Anti-GST Mouse Monoclonal Antibody (1:5000 dilution; TransGen Biotech, Catalog # HT601-01, Lot # Q20901), ProteinFind® Anti-His Mouse Monoclonal Antibody (1:5000 dilution; TransGen Biotech, Catalog # HT501-01, Lot # Q21105), ProteinFind® Anti-GFP Mouse Monoclonal Antibody (1:5000 dilution; TransGen Biotech, Catalog # HT801-01, Lot # O21209), ProteinFind® Anti-c-Myc Mouse Monoclonal Antibody (1:5000 dilution; TransGen Biotech, Catalog # HT101-01, Lot # P21018), Anti β-Actin Mouse Monoclonal antibody (1:5000 dilution; CWBIO, Catalog # CW0264M, Lot # 01265/35721,Clone # 6D1)and the secondary antibody of Goat Anti-Mouse IgG (H&L)-HRP Conjugated (1:5000 dilution; EASYBIO, Catalog # BE0102-100) were used in this study.

Validation

Each the primary antibody has been validated for the application used in the manuscript.
1. ProteinFind® Anti-GFP Mouse Monoclonal Antibody is a purified monoclonal antibody that exhibits high specificity in recognizing the GFP tag at either the C-terminus or N-terminus of recombinant proteins; The antibody can application for western blot (WB), immunoprecipitation (IP), ELISA (EIA); Citation: Li, Y., Zhang, Z., Chen, J. et al. Stella safeguards the oocyte methylome by preventing de novo methylation mediated by DNMT1. Nature 564, 136–140 (2018). https://doi.org/10.1038/s41586-018-0751-5.
2. ProteinFind® Anti-c-Myc Mouse Monoclonal Antibody is a purified monoclonal antibody that detects recombinant proteins containing the c-Myc (EQKLISEEDL) epitope tag; The antibody can application for western blot (WB), immunoprecipitation (IP), ELISA (EIA), Immunofluorescence (IF); Citation: Sun, X., Peng, X., Cao, Y. et al. ADNP promotes neural differentiation by modulating Wnt/β-catenin signaling. Nat Commun 11, 2984 (2020). https://doi.org/10.1038/s41467-020-16799-0.
3.ProteinFind® Anti-GST Mouse Monoclonal Antibody is a purified monoclonal antibody against yeast Y258 GST recombinant proteins that detects GST fusion proteins; The antibody can application for western blot (WB), immunoprecipitation (IP), ELISA (EIA); Ciation: Zhang L, Yu Z, Xu Y, et al. Regulation of the stability and ABA import activity of NRT1.2/NPF4.6 by CEPR2-mediated phosphorylation in Arabidopsis. Mol Plant, 14(4), 633-646 (2021). https://doi.org/10.1016/j.molp.2021.01.009.
4.ProteinFind® Anti-His Mouse Monoclonal Antibody is a purified monoclonal antibody that detects recombinant proteins containing the 6×His (HHHHHH) epitope tag; The antibody can application for western blot (WB), immunoprecipitation (IP), Immunofluorescence (IF), ELISA (EIA); Ciation: Li, Y., Shen, H., Zhang, R. et al. Immunoglobulin M perception by FcμR. Nature 615, 907–912 (2023). https://doi.org/10.1038/s41586-023-05835-w.
5.Anti β-Actin Mouse Monoclonal Antibody is a purified monoclonal antibody that exhibits high specificity in recognizing full-length ACTIN protein; The antibody can application for western blot (WB), ELISA (EIA); Citation: Wang, B., Zhang, H., Huai, J. et al. Condensation of SEUSS promotes hyperosmotic stress tolerance in Arabidopsis. Nat Chem Biol 18, 1361–1369 (2022). https://doi.org/10.1038/s41589-022-01196-z.

Eukaryotic cell lines

Policy information about cell lines and Sex and Gender in Research

Cell line source(s)

Yeast (Saccharomyces cerevisiae) strain AH109 (Weidi biotechnology, China, YC1010).

Authentication

None of the cell lines used were authenticated.

Mycoplasma contamination

None of the cell lines used were authenticated.

Commonly misidentified lines
(See ICLAC register)

No misidentified lines were used in this study.

Dual use research of concern

Policy information about dual use research of concern

Hazards

Could the accidental, deliberate or reckless misuse of agents or technologies generated in the work, or the application of information presented in the manuscript, pose a threat to:

No	Yes	
☒	☐	Public health
☒	☐	National security
☒	☐	Crops and/or livestock
☒	☐	Ecosystems
☒	☐	Any other significant area

Experiments of concern

Does the work involve any of these experiments of concern:

No	Yes	
☒	☐	Demonstrate how to render a vaccine ineffective
☒	☐	Confer resistance to therapeutically useful antibiotics or antiviral agents
☒	☐	Enhance the virulence of a pathogen or render a nonpathogen virulent
☒	☐	Increase transmissibility of a pathogen
☒	☐	Alter the host range of a pathogen
☒	☐	Enable evasion of diagnostic/detection modalities
☒	☐	Enable the weaponization of a biological agent or toxin
☒	☐	Any other potentially harmful combination of experiments and agents
